# Acetylcholinesterase inhibitor insecticides related acute poisoning, availability and sales: trends during the post-insecticide-ban period of Anuradhapura, Sri Lanka

**DOI:** 10.1186/s12199-018-0716-1

**Published:** 2018-06-26

**Authors:** Devarajan Rathish, Suneth Agampodi, Channa Jayasumana

**Affiliations:** 1 Department of Pharmacology, Faculty of Medicine and Allied Sciences, Rajarata University of Sri Lanka, Saliyapura, Sri Lanka; 2 Department of Community Medicine, Faculty of Medicine and Allied Sciences, Rajarata University of Sri Lanka, Saliyapura, Sri Lanka

**Keywords:** Organophosphate, Carbamate, Acetylcholinesterase inhibitors, Pesticide ban, Sri Lanka

## Abstract

**Background:**

Acetylcholinesterase inhibitor insecticides (AChEIIs) were used extensively in the agrarian region of Anuradhapura for the past few decades. As a result, the region faced a heightened risk of toxicity. Carbaryl, carbofuran, chlorpyrifos, dimethoate, and fenthion were the five hazardous AChEIIs banned from Anuradhapura in 2014. Assessment of post-ban trends in acute poisoning will reveal the impact of the ban. Data on availability and sales of remaining AChEIIs will guide towards preventive measures against related toxicities.

**Methods:**

Cross-sectional surveys were conducted at Anuradhapura district of Sri Lanka. Details related to acute AChEII poisoning were sorted from the Teaching Hospital Anuradhapura. Main insecticide vendors in Anuradhapura were surveyed to find information on availability and sales of AChEIIs. Chi-square for goodness of fit was performed for trends in acute poisoning and sales.

**Results:**

Hospital admissions related to acute AChEII poisoning have declined from 554 in 2013 to 272 in 2017. Deaths related to acute AChEII poisoning have declined from 27 in 2013 to 13 in 2017. Sales of all five banned AChEIIs had reduced by 100%. Sales of the remaining AChEIIs were declining, except for acephate, phenthoate, and profenofos. However, one of the top selling, most frequently abused carbosulfan, had the highest risk of toxicity. Chi-square for goodness of fit showed a significance (*P* < 0.001) between the trends of hospital admissions for acute AChEII poisoning and the sales related to AChEIIs.

**Conclusions:**

Hospital admissions related to acute poisoning was declining along with the overall sales of remaining AChEIIs, during the post-AChEII ban period. Nevertheless, future vigilance is needed on the remaining AChEIIs to predict and prevent related toxicities.

**Electronic supplementary material:**

The online version of this article (10.1186/s12199-018-0716-1) contains supplementary material, which is available to authorized users.

## Background

Acetylcholinesterase inhibitor insecticides (AChEIIs) include organophosphate insecticides (OPIs) and carbamates [1]. AChEIIs are commonly used in agriculture and in deliberate self-harm. Acute poisoning causes an accumulation of acetylcholine at nerve endings, leading to clinical effects on the central and peripheral nervous systems [2]. Chronic toxicity mainly involves disturbances to the endocrine, reproductive, and nervous systems [3]. In addition, these agents can have hazardous effects on the environment such as effects to the aquatic life [4]. Globally, 3 million episodes of pesticide poisoning occur out of which nearly 250,000 deaths occurring every year [5]. Asia had a case fatality rate of 5–20% for deliberate ingestion of OPIs [6]. Pesticide ingestion is the commonest method of non-fatal deliberate self-harm in Sri Lanka [7, 8]. Banning of highly hazardous pesticides is thought to be the most effective way of reducing morbidity and mortality related to self-poisoning in rural Asia [9].

In 2001, there was a mass-scale, island-wide, legal ban on pesticides by the Sri Lankan government. However, it took more than a decade to witness the next such ban [10]. Dimethoate and fenthion (both OPIs) faced a ban in 2014. The same year witnessed a regional restriction for sales of carbaryl, carbofuran, and chlorpyrifos in five agricultural districts of Sri Lanka including Anuradhapura. The other districts were Polonnaruwa, Kurunegala, Moneragala, and Badulla [10]. Chlorpyrifos is an OPI, whereas carbaryl and carbofuran are carbamates. The ban of registration in Sri Lanka for chlorpyrifos, carbaryl, and carbofuran eventually occurred in 2016 [10]. Nevertheless, the regulations for the ban on these agents were taking place from 2013 [10]. Carbofuran is classified as highly hazardous, whereas chlorpyrifos, carbaryl, dimethoate, and fenthion are classified as moderately hazardous [11].

There is valid evidence, from previous Sri Lankan studies, for the reduction of morbidity and mortality of deliberate self-harm in relation to a pesticide ban. These studies were related to pesticide bans executed before the year 2014. Roberts et al. reveal that the Sri Lankan pesticide regulation had led to a reduction in mortality during 1986 to 2000 amidst a rise in morbidity of pesticide-related deliberate self-harm [12]. Gunnell et al. stress, with evidence from Sri Lankan data (1975 to 2005), that import controls on most toxic pesticides are important to curtail incidence of self-poisoning [13]. Eddleston et al. had addressed a limitation in benefit when the ban was restricted for a particular region. He refers to the effects of restricted ban of dimethoate and fenthion in Polonnaruwa district during the year 2003 [14]. Subsequently, Knipe et al. finds that the 3-year-phased ban of paraquat (2009–2011), dimethoate and fenthion (2008–2010) in Sri Lanka had resulted in a decrease in pesticide-related suicide mortality [15]. The above studies show how a country, which once had higher rates of pesticide self-poisoning, managed to curtail the issue through regulations of pesticides.

The impact of the 2014 insecticide ban on acute AChEII poisoning is yet to be evaluated. Moreover, availability and sales of remaining AChEIIs need to be surveyed to further reduce morbidity and mortality related to poisoning. This study aims at finding the trends in acute AChEII poisoning of Anuradhapura during the post-insecticide ban period. In addition, it focuses on the availability and sales of remaining AChEIIs during the same period.

## Methods

### Study setting

The cross-sectional surveys were conducted in Anuradhapura, Sri Lanka. Ninety-five percent of the households in Anuradhapura district of Sri Lanka belong to the rural sector [16]. Fifty-five percent of the population are involved in agriculture [17]. The mean monthly household income of the district is 367 USD, compared to 391 USD for Sri Lanka [18]. Anuradhapura is known for the use and abuse of pesticides, and it had a case fatality rate of 5.8% for deliberate ingestion of OPIs [19].

### Component 01: admissions and deaths related to acute AChEII poisoning from 2013 to 2017 at Teaching Hospital Anuradhapura, Sri Lanka

The cross-sectional survey was conducted at the teaching hospital of Anuradhapura which provides free universal health care. It is the only tertiary care hospital available for the entire North-central province of Sri Lanka, and it is maintained by the government. A previous study revealed adequate availability of atropine (an antidote for acute AChEII poisoning) in primary care institutions [20]. However, it is noted that pralidoxime (an antidote for acute OPI poisoning) is listed as an essential medicine only for secondary and tertiary care institutions [21]. In addition, ventilator facilities, for respiratory failure due to AChEII poisoning, are scarce in peripheral hospitals. Due to the above reasons, majority of acute AChEII poisoning patients visit the teaching hospital directly or by transfer from other peripheral hospitals of Anuradhapura. This makes it the only low-cost option for patients of Anuradhapura to seek specialized care for acute AChEII poisoning.

Data on annual admissions and deaths (2013 to 2017) related to acute AChEII poisoning among adult medical ward patients were retrieved from the record room of the teaching hospital of Anuradhapura. Collected data were entered to a Microsoft Excel sheet. Data were presented as graphical trends over the 5 years in focus. These trends were related to the timing of specific Sri Lankan AChEII regulation (2014). The outcomes of interest were overall admissions, male admissions, female admissions, deaths, and deaths per admission related to acute AChEII poisoning. Chi-square for goodness of fit was performed to find a significance (*P* < 0.05) in the trends related to acute poisoning. Respective averages of the above parameters were used as the expected value. The commonly used AChEIIs for self-poisoning were found through a sub-group analysis using the data from July to December 2017 of male patients aged ≥ 18 years.

### Component 02: availability of AChEIIs and its possible toxicities, Anuradhapura, Sri Lanka, 2017

The cross-sectional survey was conducted during the first week of August 2017. Details on availability of AChEIIs were obtained from the main vendor of the following divisional secretariat divisions of Anuradhapura district: Nuwaragam Palata East (Anuradhapura town area), Glenbindunuwewa, Kahatagasdigiliya, Medawachchiya, Padaviya, and Thambuttegama. The main vendor of Nuwaragam Palata East supplies goods for the entire Anuradhapura district (wholesale and retail). Thambuttegama is the main division for vegetable farming; therefore, the use of AChEIIs is assumed to be high. Medawachchiya and Padaviya are the main divisions for paddy cultivation. Glenbindunuwewa and Kahatagasdigiliya are the main divisions for corn and soya cultivation. Inclusion of all major divisions of cultivation would yield optimum data. Availability of AChEIIs was also checked at the main wholesale dealer located near the economic center of Dambulla secretariat division of the Matale district. This is around 65 km away from Anuradhapura town, making it a 1 ½-hour travel. It is a meeting place for several farmers and thereby an important sales outlet for AChEIIs near Anuradhapura.

Details of active ingredients of all available insecticides were collected from each vendor. Collected data were entered to a Microsoft Excel sheet. Details on AChEIIs were separated for further analysis. Availability as percentage was described by active ingredients and by secretariat divisions. The “World Health Organization recommended classification of pesticides by hazard and guidelines to classification 2009” [11] and the United Nations “globally harmonized system of classification and labeling of chemicals” from the PubChem website [22] were used to find the toxicity profile of each available AChEIIs.

### Component 03: sales of AChEIIs from 2015 to 2017 at Anuradhapura, Sri Lanka

The main dealer of Anuradhapura supplies AChEIIs (whole- and retail sales) for the entire Anuradhapura district. Computerized annual sales information for AChEIIs from years 2015 to 2017 was available and retrieved. The above reasons helped to obtain optimum data with the best representation for the entire district. Collected data were entered to a Microsoft Excel sheet. Sales of AChEIIs were recorded in either kilograms or milliliters according to the available preparations. However, a uniform unit was needed for comparison; therefore, a conversion was done using the strength of each preparation. For example, diazinon granules had a strength of 50 g/kg. The total sale of diazinon in kilograms was multiplied by 50. Profenofos had strength of 500 g/l. The total sale in milliliters was converted to liters and then multiplied by 500. By the above conversion, the total sale of active ingredient was calculated in grams. Data were presented for the 3 years in focus. Comparison was made between the annual sale of each AChEII. In addition, comparison was made with the annual admissions related to acute AChEII poisoning.

## Results

### Admissions and deaths related to acute AChEII poisoning from 2013 to 2017 at Teaching Hospital Anuradhapura, Sri Lanka

**Fig. 1:**
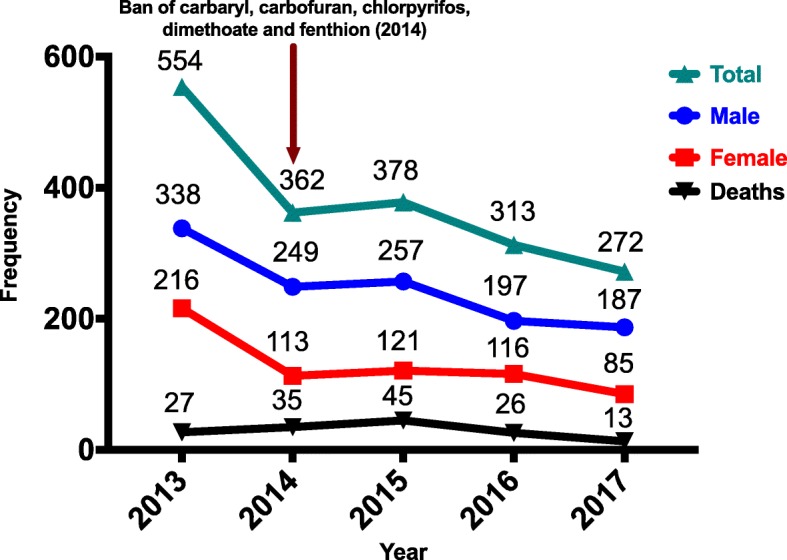
Admissions and deaths related to acetylcholinesterase inhibitor insecticide poisoning by year, AChEII study, Anuradhapura, 2017

**Fig. 2:**
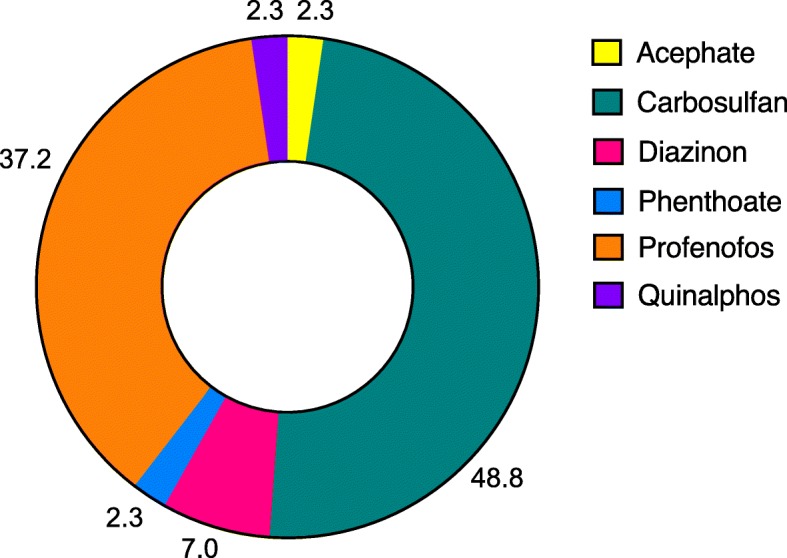
Acetylcholinesterase inhibitor insecticides used in self-poisoning among adult males from July to December 2017 by percentage, AChEII study, Anuradhapura, 2017

The overall, male, and female admissions and deaths for acute AChEII poisoning were 554, 338, 216, and 27, respectively, in 2013. This has dropped to 272, 187, 85, and 13, respectively, in 2017 showing a downward trend over the last 5 years (Fig. 1). By percentage, the drop is 51, 45, 61, and 52% for the overall, male, and female admissions and deaths, respectively, from 2013 to 2017. Deaths as a percentage of admissions were 4.9, 9.7, 11.9, 8.3, and 4.8% for years 2013 to 2017, respectively. There was a significant difference (*P* < 0.001) in the trends of overall admissions, male admissions, female admissions and deaths related to acute AChEII poisoning (Additional file 1). However, trends in deaths per admission failed to show a significance (*P* = 0.314). Records of male patients, aged ≥ 18 years from July to December 2017, had 43 who were identified and recorded with the specific AChEII used for self-poisoning. Among those identified, 48.8% (21/43) had consumed carbosulfan, making it the commonest AChEII used in self-poisoning (Fig. 2).

### Availability of AChEIIs in Anuradhapura, Sri Lanka, 2017

**Fig. 3:**
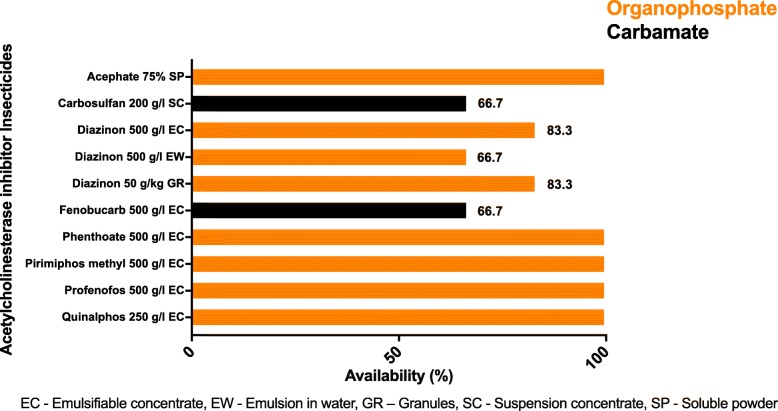
Availability of acetylcholinesterase inhibitor insecticides among the six surveyed vendors of the divisions of cultivation, AChEII study, Anuradhapura, 2017

A total of eight AChEIIs in ten different preparations were found across the six surveyed divisions of Anuradhapura (Fig. 3). Carbosulfan and fenobucarb were the only carbamates available, and the rest were OPIs. Diazinon was found in three different preparations (emulsifiable concentrate, emulsion in water, and granules). Nuwaragam Palata East and Thambuttegama divisions had all ten AChEII preparations. However, the availability of the ten AChEII preparations was 90, 80, 80, and 70% at Medawachchiya, Padaviya, Glenbindunuwewa, and Kahatagasdigiliya divisions, respectively. The Dambulla (Matale district) division also had all ten preparations of AChEIIs. Acephate, carbosulfan, diazinon, profenofos, and quinalphos were found in all six divisions (Fig. 3 and Additional file 1).

### Annual sales of AChEIIs at Anuradhapura, Sri Lanka, 2015 to 2017

**Fig. 4:**
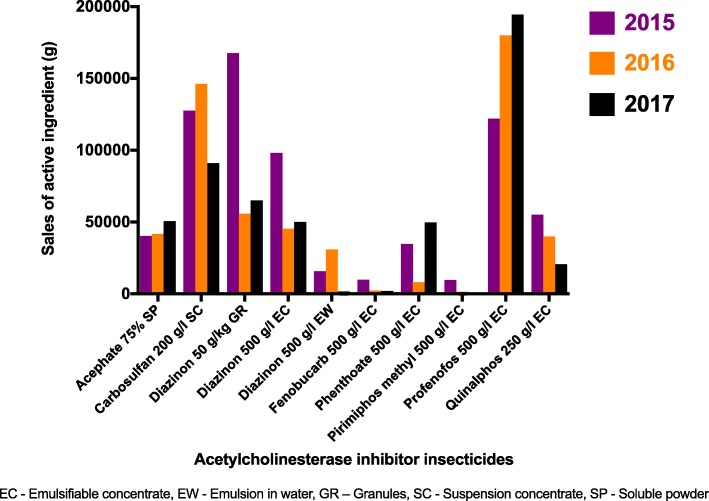
Sales of acetylcholinesterase inhibitor insecticides by active ingredient, AChEII study, Anuradhapura, 2017

Data for sales of eight AChEIIs in ten different preparations were found. These were the same preparations found from the component 02 of the survey. The top three sales of active ingredients were seen with diazinon (total 281,575 g), carbosulfan (127,505 g), and profenofos (122,041 g), respectively, for the year 2015. It was profenofos (179,988 g), carbosulfan (146,125 g), and diazinon (total 132,169 g), respectively, for 2016; profenofos (194,469 g), diazinon (total 115,850 g), and carbosulfan (91,070 g), respectively, in 2017 (Fig. 4 and Additional file 1).

**Fig. 5:**
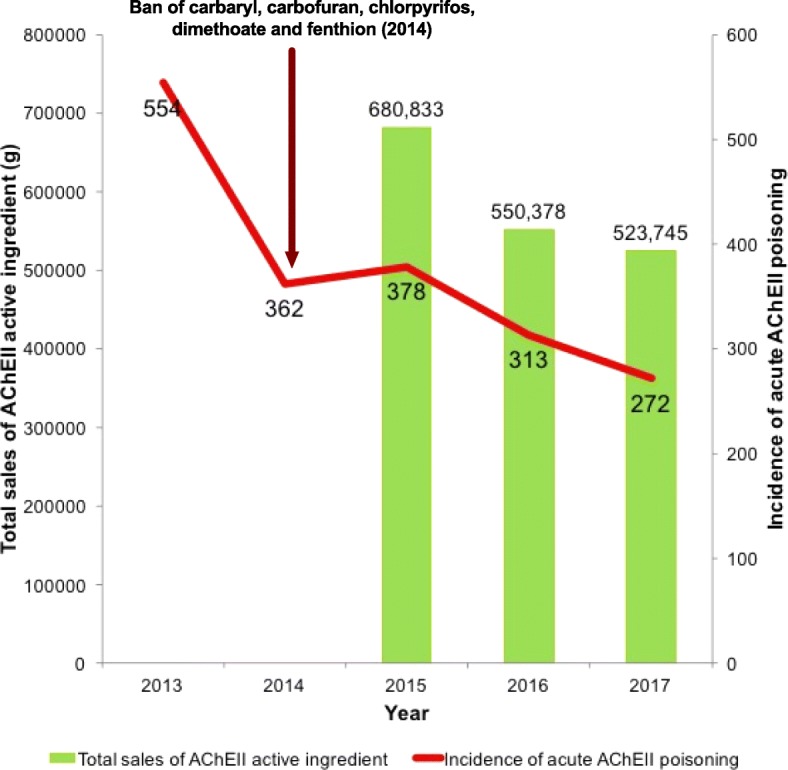
Total sales of acetylcholinesterase inhibitor insecticides compared with the admissions of acute acetylcholinesterase inhibitor insecticide poisoning by year, AChEII study, Anuradhapura, 2017

In comparison to 2015, the sales of the following have increased in 2017: acephate (26%), phenthoate (43%), and profenofos (59%). The rest showed a decrease in sales from 2015 to 2017 (Fig. 4). Pirimiphos-methyl has shown the highest drop by 99%. Overall, the sales of AChEIIs (active ingredient) have reduced from 680,833 g in 2015 to 523,745 g in 2017 showing a drop by nearly 23%. There was a significant difference (*P* < 0.001) in the trends of sales related to AChEIIs (Additional file 1). In addition, there is a decline in admissions along with the sales from 2015 to 2017 (Fig. 5). A statistical test for correlation was inappropriate as data was available only for 3 years.

## Discussion

In line with previous similar Sri Lankan studies, the findings of this survey provide evidence for reduction in morbidity related to acute AChEII poisoning during the post-AChEII ban period [12–15]. Carbosulfan, the available most toxic compound, especially via oral ingestion, was most frequently abused. Carbosulfan was available in all six surveyed divisions of Anuradhapura and was one of the top three selling AChEIIs. Surprisingly, the sales of 5 out of 8 remaining AChEIIs have shown to reduce over the 3-year post-AChEII ban period. However, the sale of profenofos was increasing and it is the second commonest agent used in self-poisoning. The fall in the overall sales of AChEIIs could be attributed to the decline in cultivation. The decline in cultivation was partly due to lack of rain fall for the district from the beginning of 2015 to mid-2017 [23] and the unaffordable rise in prices of fertilizers [24]. These have made the farmers to choose alternative means for their living, causing a drop in sales of insecticides.

The fall in admissions related to acute AChEII poisoning could be due to the following factors: fall in sales of AChEIIs in Anuradhapura district (Figs. 4 and 5 and Additional file 1), less hazardous nature of the remaining AChEIIs in comparison to the banned AChEIIs, change of methods used for deliberate self-harm (from insecticides to medicinal products) [19], and awareness of private pesticide vendors on restricting sales of pesticides for deliberate self-harm [25]. There was no evidence that safe storage of insecticides at home would reduce deliberate self-harm. Nevertheless, banning of highly hazardous insecticides is thought to be more effective in rural Asia for prevention of deliberate self-harm [9]. The present survey revealed no significance in the trends for deaths per admission during the 5-year period in focus. Mortality rates depend not only on the availability of the toxic agent but also on the amount ingested, time taken to reach the hospital, co-morbidities, availability of treatment modalities, and the rebound effect seen with AChEII poisoning. Therefore, insecticide banning alone cannot be expected to significantly reduce mortality rates.

In a setting like Anuradhapura, where the use and abuse of AChEIIs is common, it is essential to find the available AChEIIs in the market. This would help us to understand its potential toxic effects, so that medical professionals and the healthcare system are ready for the prevention, prediction, and management of AChEII-related poisoning. According to the survey, the following are the remaining AChEIIs at Anuradhapura district: acephate, carbosulfan, diazinon, fenobucarb, phenthoate, pirimiphos-methyl, profenofos, and quinalphos (Figs. 3 and 4 and Additional file 1). Dambulla division of the Matale district too had all of the above AChEIIs. Therefore, a restricted ban to a particular district might not be effective because the insecticides could be purchased by traveling to the neighboring district.

All available AChEIIs fell under class II of acute toxicity (moderately hazardous) [11]. A pesticide is classified as *moderately hazardous* when it has a lethal dose of 50–2000 mg/kg and 200–2000 mg/kg, via oral and dermal routes, respectively, for a rat [11]. According to “the globally harmonized system of classification and labeling of chemicals” from the PubChem website [22], the toxicity profile of the eight available AChEIIs is summarized and compared with that of the 2014-banned AChEIIs in Table 1. All available AChEIIs showed a risk of acute toxicity via oral route. Carbosulfan and quinalphos were classified as dangerously toxic via oral route. A dangerous level of toxicity was predicted with carbosulfan, phenthoate, profenofos, and quinalphos when inhaled. Profenofos was predicted to be dangerously toxic via dermal route too. Skin sensitization could occur with carbosulfan, diazinon, and profenofos. Acephate, carbosulfan, and quinalphos could produce serious eye damage or eye irritation. Risk of reproductive toxicity was seen with acephate and diazinon. Risk of specific target organ toxicity (both by single and repeated exposure) was seen with acephate, carbosulfan, and diazinon. All available AChEIIs showed risk of acute and long-term toxicity for aquatic life (Table 1). Out of the toxicity risks mentioned, only the risk of serious eye damage/eye irritation was absent for diazinon and only the risks of reproductive toxicity were absent for carbosulfan.

**Table 1:** Toxicity profile of available and banned acetylcholinesterase inhibitor insecticides, AChEII study, Anuradhapura, 2017

AChEII	Acute toxicity			Sensitization of the skin	Serious eye damage/eye irritation	Reproductive toxicity	Specific target organ toxicity		Toxic to aquatic life	
	Oral	Dermal	Inhalation				Single exposure	Repeated exposure	Acute hazard	Long-term hazard
Acetylcholinesterase inhibitor insecticides available at Anuradhapura in 2017										
Acephate	Warning	NA	NA	NA	Warning	Warning	Danger	Warning	Present	Present
Carbosulfan	Danger	Warning	Danger	Warning	Warning	NA	Danger	Warning	Warning	Warning
Diazinon	Warning	Warning	Warning	Warning	NA	Warning	Danger	Danger	Warning	Warning
Fenobucarb	Warning	NA	NA	NA	NA	NA	NA	NA	Warning	Warning
Phenthoate	Warning	Warning	Danger	NA	NA	NA	NA	NA	Warning	Warning
Pirimiphos-methyl	Warning	NA	NA	NA	NA	NA	NA	NA	Warning	Warning
Profenofos	Warning	Danger	Danger	Warning	NA	NA	NA	NA	Warning	Warning
Quinalphos	Danger	Warning	Danger	NA	Danger	NA	NA	NA	Warning	Warning
Acetylcholinesterase inhibitor insecticides banned at Anuradhapura in 2014										
Carbaryl	Danger	NA	Warning	NA	NA	NA	NA	NA	Warning	Warning
Carbofuran	Danger	Present	Danger	NA	NA	Warning	Danger	Danger	Warning	Warning
Chlorpyrifos	Danger	Warning	Danger	NA	Warning	NA	NA	NA	Warning	Warning
Dimethoate	Danger	Danger	NA	NA	Warning	NA	Danger	Warning	Present	Present
Fenthion	Warning	Warning	Danger	NA	NA	Warning	NA	Danger	Warning	Warning

*AChEIIs* acetylcholinesterase inhibitor insecticides, *NA* not available

Carbosulfan, diazinon, and profenofos have remained as the top three AChEIIs, by amount of active ingredient sold, for the last 3 years. In addition, those were the top three agents abused too. It is alarming to note that diazinon and carbosulfan had the highest risk of acute, chronic, and environmental toxicities among the available AChEIIs (Table 1). The survey reveals a pattern of increase in abuse of the remaining most toxic substances which may have been enhanced by its higher availability and sales.

Sales details for AChEIIs were retrievable only from the main dealer of AChEIIs at Anuradhapura. The complete annual sales details were computerized for years 2015, 2016, and 2017. In addition, the admissions for acute AChEII poisoning were retrieved only from the teaching hospital of Anuradhapura because majority of these cases from other hospitals of the district were eventually transferred to the teaching hospital. Identification of the specific AChEII used in self-poisoning is challenging and needs an in-depth interview with the patient or caretaker, if the specimen was not to be found. However, we used the recorded identification of AChEIIs among male patients, aged ≥ 18 years to find the common agents abused. The above selections were the best possible representation for the district which had yielded optimum data. This survey provides evidence for the effectiveness of AChEII ban in reducing morbidity of acute AChEII poisoning. Further studies, to monitor the toxic effects and the incidence of acute toxicity related to the remaining AChEIIs, are essential to prevent health and environmental hazards.

## Conclusions

The post-AChEII ban period shows a reduction in admissions of acute poisoning and a decline in the overall sales of remaining AChEIIs. Nevertheless, the highest selling agents at present are also the top agents abused. Vigilance is needed on the remaining AChEIIs to predict and prevent related toxicities.

## Additional file

Additional file 1: Admissions, availability, and sales related to acute AChEII poisoning, AChEII study, Anuradhapura, 2017. This provides the data of the entire survey and the results of chi-square for goodness of fit. (XLS 50 kb)

## Abbreviations

AChEIIs: Acetylcholinesterase inhibitor insecticides
OPI: Organophosphate insecticides
USD: United States dollars
WHO: World Health Organization
